# Escape response and perch-site choice in shrikes: effects of human disturbance in Europe and Southeast Asia

**DOI:** 10.7717/peerj.21506

**Published:** 2026-06-30

**Authors:** Zbigniew Kasprzykowski, Artur Goławski, Cezary Mitrus, Santi Xayyasith, Przemysław Obłoza

**Affiliations:** 1 Faculty of Sciences, University of Siedlce, Siedlce, Poland; 2 Department of Vertebrate Ecology and Paleontology, Institute of Environmental Biology, University of Environmental and Life Sciences, Wrocław, Poland; 3 Faculty of Environmental Sciences, National University of Laos, Vientiane, Laos

**Keywords:** Antipredator behaviour, Human disturbance, Vigilance, Flight initiation distance

## Abstract

**Background:**

Anthropogenic pressure, including hunting and habitat modification, is a major driver of behavioral variation in birds, particularly in regions with differing conservation regimes and human disturbance levels. Flight initiation distance (FID) is a widely used metric of antipredator behavior that reflects both immediate risk perception and longer-term behavioral adjustments to human activity. This study compares habitat choice and escape responses of two closely related species, the Red-backed Shrike (*Lanius collurio*) and the Brown Shrike (*Lanius cristatus*), occurring in regions with contrasting levels of human pressure: Central Europe (Poland) and Southeast Asia (Laos). We tested whether the two species differ in their use of perching sites relative to roads and built-up areas, and whether FID is shaped by proximity to human infrastructure and species identity.

**Methods:**

Field data were collected in spring 2024 in agricultural and peri-urban landscapes in Poland and Laos. A total of 171 perching sites were recorded (102 for Red-backed Shrikes and 69 for Brown Shrikes). For each observation, FID, starting distance (SD), perch height, and distances to the nearest dirt road, asphalt road, and built-up area were measured. Determinants of FID were examined using linear models with log-transformed response variables. Model selection was based on an information-theoretic approach using Akaike Information Criterion (AIC), and only models with Δ AIC ≤ 2 were retained for inference.

**Results:**

Perch-site choice differed significantly between the two species. Brown Shrikes occurred at greater distances from built-up areas. FID was significantly greater in Brown Shrikes than in Red-backed Shrikes. Distance to the nearest built-up area was negatively associated with FID, while SD had a positive impact. These results indicate that shrikes inhabiting regions with higher hunting pressure exhibit stronger avoidance behavior and altered spatial responses to human infrastructure, highlighting the role of regional anthropogenic context in shaping antipredator behavior.

## Introduction

We live in a period of accelerated global biodiversity loss, and declining animal populations have profound implications for ecosystem functioning and human well-being (Dirzo et al., 2014). While behavioral plasticity and habituation may mitigate some effects of human disturbance (Procko et al., 2023; Lee et al., 2024), direct anthropogenic mortality continues to exert strong and often additive pressures on wildlife populations (Loss, Will & Marra, 2015; Baral et al., 2022). Hunting of wild animals is one of the oldest and most widespread activities across human populations worldwide (Alves et al., 2009). Among the most frequently hunted groups, both in terms of species diversity and the number of individuals, are mammals and birds (Nijman, 2010; Alves, de Lima & Araujo, 2013; Bhupathy et al., 2013; Roldán-Clarà et al., 2014). Throughout history, wild birds have been an important source of food for humans, from prehistoric subsistence hunting to modern exploitation across regions (*e.g.*, Yeomans & Mazzucato, 2024; Commerçon, Zhang & Solomon, 2021).

From a traditional perspective and in contrast to many European countries, wild birds in Southeast Asia are often viewed as a resource to be exploited rather than subjects for conservation (Zhang, Hua & Sun, 2008). Each year, vast numbers of birds are sold openly at wildlife markets in this region (Nijman, 2010; Root et al., 2006). They are traded live as pets and collectors’ items, or dead for food, medicine, ornaments, and trophies (Burivalova et al., 2017). Beyond hunting with firearms, birds face broader pressures from trapping, which often results in the incidental killing of non-target species (Angkaew et al., 2022), as well as the use of playback calls, scare tactics, or slingshots (Dai & Hu, 2017; Katuwal et al., 2023). This is of particular concern because Southeast Asia has a higher proportion of bird species categorized on the Red List as globally threatened (Sodhi et al., 2010). Located within biodiversity ‘hotspots,’ this region possesses the highest average proportion of native endemic birds among tropical areas (Myers et al., 2000). In contrast, many bird species (including shrikes) in most European countries are effectively protected by national laws and European Union regulations, leading to much lower levels of illegal hunting, especially in Central Europe (CABS, 2010; Brochet et al., 2019). However, in the Mediterranean region, the hunting of migratory birds remains a significant and unresolved problem despite EU legislation (Brochet et al., 2019). Birds nesting in Central or Northern Europe are therefore exposed to increased human pressure, primarily during migration through the Mediterranean.

Monitoring bird populations near human settlements is essential for linking population trends to hunting pressure and informing conservation actions (Xayyasith, Douangboubpha & Chaiseha, 2020). Such efforts also track the significant transformations of traditional rural landscapes into mosaics of agricultural land, settlements, and infrastructure (DeFries et al., 2010). Mitigating the threats of hunting and the wildlife trade requires a thorough understanding of species ecology, including the factors that influence avian wariness. Flight initiation distance (FID) serves as a robust behavioral indicator to quantify responses to approaching humans or other potential predators (Stankowich & Blumstein, 2005; Cooper Jr & Wilson, 2007). Birds typically respond to an approaching threat by fleeing at a distance that optimizes the trade-off between the costs of flight and the benefits of remaining (Cooper Jr & Frederick, 2007). Importantly, recent research indicates that FID is not merely a reaction to immediate threats; it also reflects broader behavioral adjustments to ecological and environmental conditions—such as predation risk—which are themselves modulated by anthropogenic factors (Díaz et al., 2021; Díaz & Møller, 2023; Shuai et al., 2024). FID is closely linked to synanthropization, as human proximity and associated environmental disturbances often lead to shorter flight distances (Møller, 2008; Samia et al., 2017). For instance, FID in response to humans is typically shorter in urban areas than in rural ones (Morelli et al., 2022). Furthermore, some species exhibit distinct FID responses to the presence of vehicles and human-operated machinery (McLeod et al., 2013; Kasprzykowski & Golawski, 2025).

This study tests two key hypotheses regarding the responses of Brown Shrikes (*Lanius cristatus*) and Red-backed Shrikes (*Lanius collurio*) to human disturbance. Although these species occupy largely separate geographic ranges, they are closely related both morphologically and ecologically, making them ideal candidates for comparative analysis (Lefranc, 2022). Shrikes are highly detectable and frequently utilized in behavioral research (Morelli et al., 2015; Golawski & Kasprzykowski, 2018; Mikula et al., 2022). Our first hypothesis concerns habitat choice in relation to infrastructure; we predicted that the two species would differ in their choice of perching sites relative to roads and settlements. Secondly, we hypothesized that FID is influenced by proximity to roads and built-up areas—where human pressure is highest—and that these responses differ between the Red-backed and Brown Shrike. Specifically, we expect that Red-backed Shrikes, occurring in regions of relatively low human pressure (Central Europe), will select perching sites closer to human activity and exhibit shorter FIDs than Brown Shrikes inhabiting regions with higher human pressure (Southeast Asia). Given that broad-scale measures, such as national population density, may not accurately reflect the immediate environment experienced by birds, we utilized local proxies of anthropogenic pressure: distance to roads and distance to built-up areas. By comparing these species across regions with differing hunting pressures, this study aims to clarify how anthropogenic factors shape bird behavior and habitat choice, ultimately informing more effective conservation strategies.

## Materials & Methods

### Study area

**Figure 1:**
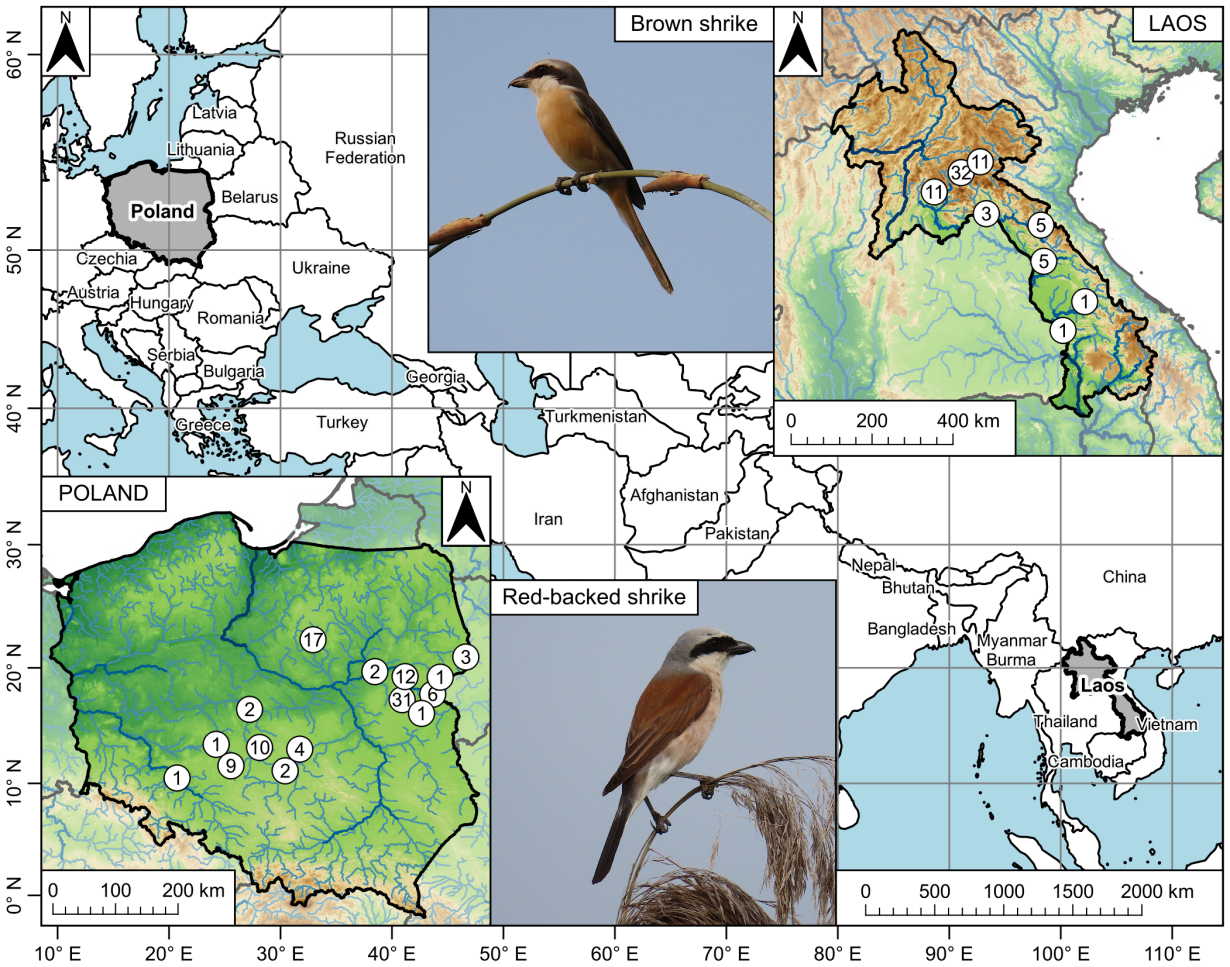
Map of the study areas in Poland and Laos. White dots indicate shrike locations (sample sizes provided in parentheses). The map was created using QGIS v3.40.1 (https://qgis.org/; GNU General Public License). Background layers were derived from the following sources: (A) Countries, 2020—Administrative Units—Dataset (EUROSTAT; CC BY 4.0); (B) the “SRTM30 colored” Digital Elevation Model (DEM) from terrestris.de and NASA EOSDIS Land Processes Distributed Active Archive Center (terrestris.de, 2024), as well as the HydroRIVERS database (Lehner & Grill, 2013).

The study was conducted in Laos and Poland (Fig. 1), two countries with distinct bird species compositions and conservation contexts. Laos is a landlocked country located in the tropical zone of Southeast Asia. Its forested landscape is predominantly mountainous, with plains and plateaus in the southern region, and it has a human population density of 28 inhabitants per km^2^ (Cosslett & Cosslett, 2018). Laos lies within the Indo-Burma biodiversity hotspot and hosts numerous rare and endemic species (Tordoff et al., 2012). However, poorly regulated hunting and illegal trade are causing severe declines in the wild populations of many species (Xayyasith, Douangboubpha & Chaiseha, 2020). Such hunting pressure can elicit faster escape responses and shifts in habitat or perch-site selection, which may, in turn, reduce foraging efficiency (Yuan et al., 2024; Beatty, Huck & Buderman, 2024). In Laos, hunting pressure is typically concentrated near villages, agricultural areas, and other human-accessible sites, where wildlife harvesting is most frequent (Johnson, Singh & Duangdala, 2003). The Lao study sites were located in landscapes with scattered settlements and cultivated areas, where opportunistic bird hunting by local people is known to occur. In contrast, Poland is located in Central Europe and is characterized by predominantly lowland terrain. Agricultural land, mainly cereal crops, covers approximately 50% of the country, while forests account for about 30%. The population density is significantly higher, at 122 inhabitants per km^2^ (GUS, 2019). In Poland, nature conservation is comprehensive, and the illegal taking of birds (particularly small passerines) is negligible (Brochet et al., 2019).

### Study species

The Red-backed Shrike (*Lanius collurio*) and Brown Shrike (*Lanius cristatus*) are small passerines that are closely related, making them ideal for comparative studies of ecological and behavioral responses to anthropogenic disturbance (Zhang et al., 2007). Both species belong to the genus *Lanius* and are part of a well-defined Palearctic clade, sharing high genetic similarity and overlapping ecological niches (Panov, 2011; Lefranc, 2022). They are highly detectable due to their “sit-and-wait” hunting strategy, regularly foraging from conspicuous perches such as bush tops, power lines, and fence posts (Yosef, 2008). Both shrikes possess comparable body sizes (20–21 cm in length; 30–35 g in mass), similar plumage structures, and trophic preferences dominated by large insects and small vertebrates (Yosef, 2008; Lefranc, 2022). Furthermore, they exhibit similar patterns of perch selection, territoriality, and predator-vigilance behavior (Yosef, 2004; Morelli et al., 2015; Golawski & Kasprzykowski, 2018). Comparative studies have repeatedly demonstrated the evolutionary stability of key foraging and anti-predator strategies within this genus (Hamao et al., 2021; Lefranc, 2022; Mikula et al., 2022), justifying the comparison of their responses to human-induced disturbances. Both species inhabit human-modified landscapes, though their association with built-up areas is typically limited (Golawski & Meissner, 2008; Golawski et al., 2026). The Red-backed Shrike is a long-distance migrant breeding across Europe and western Asia and wintering in sub-Saharan Africa (Lefranc, 2022). While many European populations are declining, the Polish population is showing a moderate increase, currently estimated at 0.74–1.1 million breeding pairs with an average density of 2.5 pairs/km^2^ (Chylarecki et al., 2018). Most of the population inhabits agricultural landscapes, breeding along woodland edges and in tree clumps, orchards, and currant plantations. In Poland, breeding Red-backed Shrikes incorporate only a small proportion of built-up areas into their territories (Golawski & Meissner, 2008). The Brown Shrike is also a long-distance migrant, breeding in Northern Asia (from Mongolia to Siberia) and wintering in South Asia, Myanmar, and the Malay Peninsula. In Laos, it is a common winter visitor from September to April, inhabiting open farmland, pastures, secondary scrub, and peri-urban gardens (Robson, 2020). The Brown Shrike is currently classified as a species of “Least Concern,” though it faces a declining global population for which no precise estimates are available (BirdLife International, 2025). Across its range, the species most frequently utilizes beach-shrub reedbeds, pastures, and urban gardens (Lefranc, 2022). Consistent with observations in Poland, Brown Shrikes have been shown to avoid built-up areas during their wintering period in Laos (Golawski et al., 2026).

### Data collection

Fieldwork was conducted in spring 2024, from 10 April to 2 May in Laos, during the wintering period or the onset of spring migration for the Brown Shrike, and from 11 May to 20 June in Poland, coinciding with the beginning of the Red-backed Shrike breeding season. Since Red-backed Shrike behavior in the presence of an observer only changes significantly when large chicks or fledglings are present (Tryjanowski & Goławski, 2004), we assumed that the two species could be compared without a significant influence of breeding status. Shrikes were located by observers walking transects along unpaved roads. Each transect was surveyed only once to ensure that no individual was measured multiple times. In Poland, the study was conducted across 15 locations (mean distance apart: 203.76 km; range: 29.5–502.9 km), and in Laos at 8 locations (mean distance apart: 250.05 km; range: 54.05–468.6 km). A total of 171 perching sites were recorded (102 for the Red-backed Shrike and 69 for the Brown Shrike). After detecting a bird, the observer walked directly toward it in a straight line at a constant speed of approximately 1.0 m/s. The distance from the starting point to the bird’s position (starting distance, SD) and the distance at which the bird initiated flight (FID) were recorded by counting steps, which were subsequently converted to meters. This method of using a consistent step length and pace has been validated in similar studies (Kasprzykowski & Golawski, 2025). Observers wore neutrally colored clothing to avoid startling the birds. Because observers did not differ substantially in height, a factor generally considered not to influence FID (*e.g.*, Guay et al., 2013), this variable was excluded from the analyses. Perch heights were measured with a tape measure (accuracy: 10 cm) after the bird had departed. For perches exceeding 3 m, height was estimated by comparing the bird’s position to the observer’s height (Wang et al., 2019; Golawski et al., 2024). Euclidean FID was calculated as the square root of the sum of the squared horizontal distance and the squared height (Blumstein, 2006). Perch sites were categorized into four types: fence, bush, tree, and power line. Distances (km) from the perching site to the nearest dirt road, asphalt road, and built-up area were measured using QGIS (QGIS Development Team, 2024; Appendix 1). Built-up areas were defined as densely developed zones, typically villages, where buildings were situated close together with minimal open space. Mapping was based on high-resolution aerial photographs (0.25 m pixel) for Poland and Google Earth satellite imagery for Laos.

### Statistical analyses

Differences between the Red-backed and Brown Shrike in their distances to the nearest dirt road, asphalt road, and built-up area were assessed using Mann–Whitney U tests. To determine the factors influencing FID, we employed linear models (LM) with a Gaussian error distribution using the *lme4* package in R (Bates et al., 2015). First, multicollinearity among quantitative variables was tested; no high values were identified (variance inflation factor, VIF < 1.5). FID was log-transformed to improve model fit, which was verified using *DHARMa* diagnostic plots (Hartig, 2024). Five continuous predictors were included: starting distance (SD), distance to the nearest dirt road (Road), distance to the nearest asphalt road (Asph), and distance to the nearest built-up area (Build). The global model also included perch category (Perch) and species (Spec) as categorical predictors: log(FID) ∼SD + Road + Asph + Build + Perch + Spec. Interactions between species and other predictors were excluded as they did not improve model fit. Models were selected using an Akaike Information Criterion (AIC) (Burnham & Anderson, 2002). All possible combinations of the global model were analyzed using the dredge function in the *MuMIn* package (Bartoń, 2020). Only models with ΔAIC≤2 were considered equally supported and retained for inference. R2 values for the candidate model sets were calculated according to Nakagawa & Schielzeth (2013). All analyses were performed in R (R Core Team, 2023). Distance values are presented as medians and quartiles, while FID is reported as mean ± CI (confidence interval). Statistical significance was set at *α*  ≤ 0.05.

## Results

**Figure 2:**
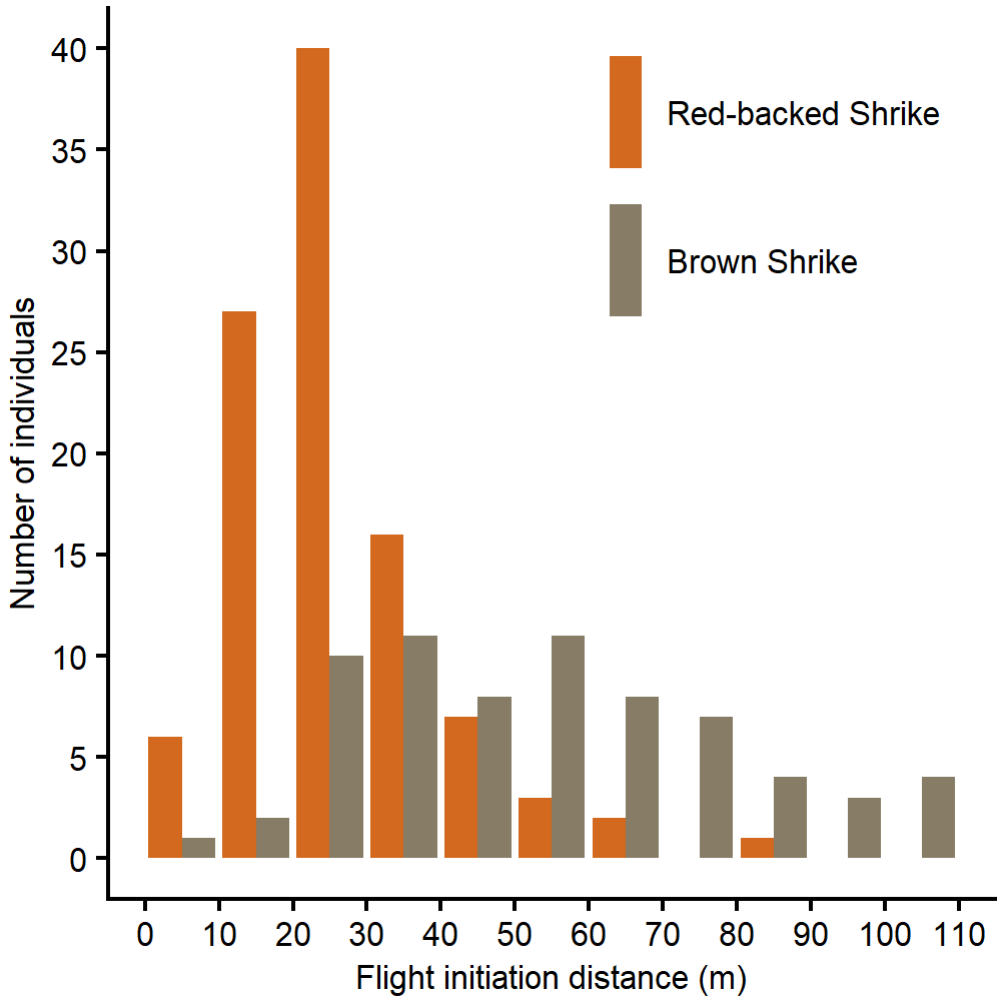
Frequency distribution of flight initiation distances (FID) for the Red-backed Shrike and Brown Shrike. Data are presented as the number of individuals within specific distance intervals.

**Figure 3:**
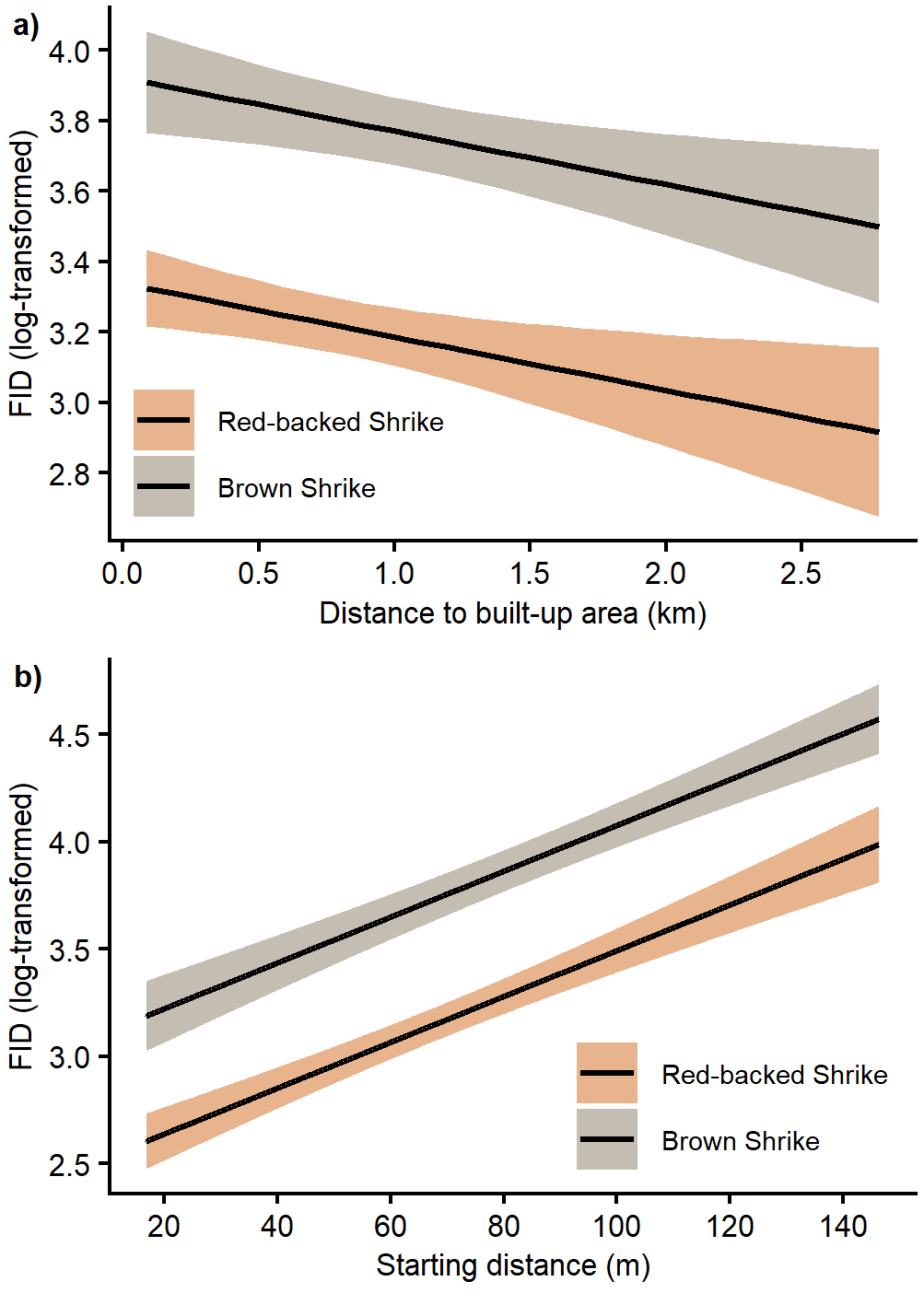
Relationship between flight initiation distance (FID) and (A) distance to the nearest built-up area and (B) starting distance for the two shrike species.

**Figure 4:**
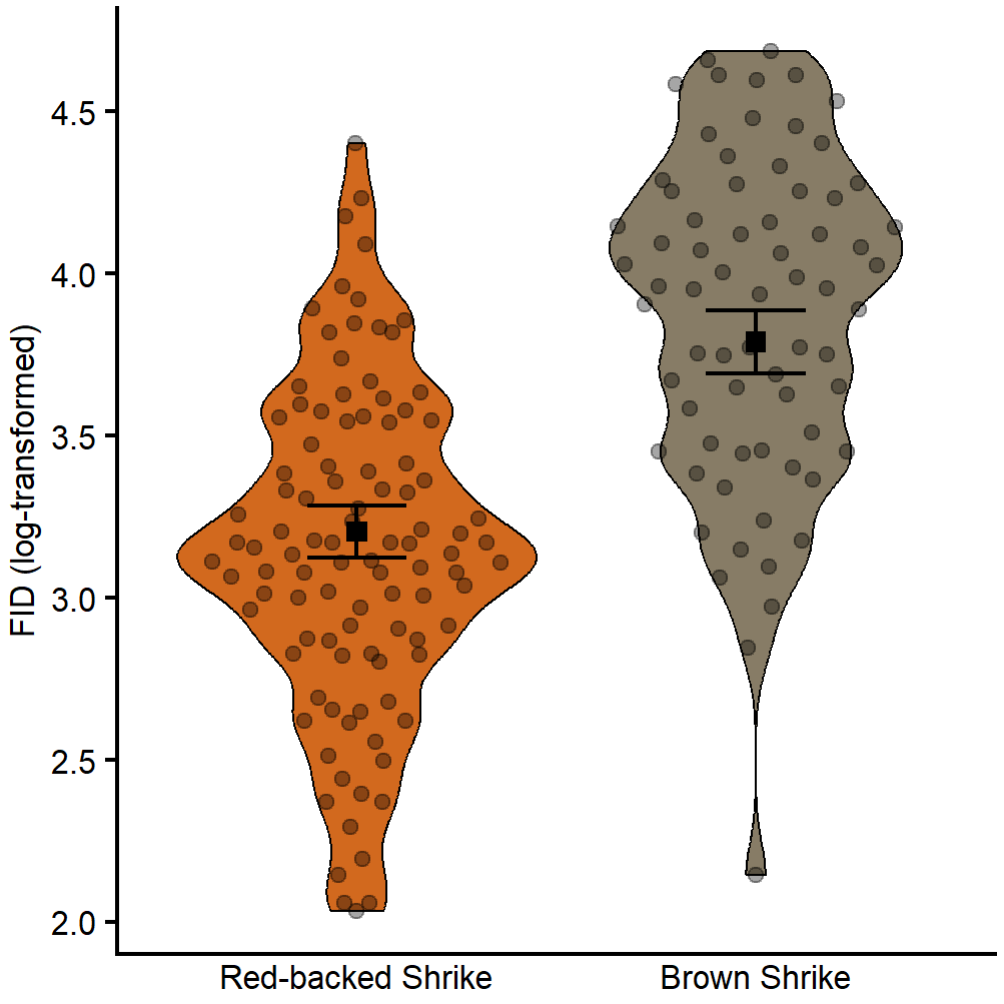
Comparison of flight initiation distances (FID) between the Red-backed Shrike and Brown Shrike. Squares represent means, error bars indicate 95% confidence intervals, and violins show the kernel density distribution. Grey dots represent raw data points.

The two shrike species differed significantly in their choice of perching sites relative to human settlements (Mann–Whitney U test: *Z* = 4.05, *p* < 0.001, *N* = 171). Brown Shrikes occurred at greater distances from built-up areas (Me = 0.97 km; IQR = 0.63–1.44) compared to Red-backed Shrikes (Me = 0.61 km; IQR = 0.40–1.01). No significant differences between species were found in the distances from perching sites to the nearest dirt road (Mann–Whitney U test: *Z* = 0.73, *p* = 0.463, *N* = 171) or the nearest asphalt road (Mann–Whitney U test: *Z* = 1.01, *p* = 0.317, *N* = 171). The Red-backed Shrike most frequently initiated flight at distances between 10 and 30 m (Mean = 26.4 m; range: 7.6–81.6 m), whereas the Brown Shrike typically flushed at distances between 20 and 60 m (Mean = 53.7 m; range: 8.5–108.6 m; Fig. 2). Modeling the factors underlying FID indicated that three out of 64 analyzed models achieved ΔAICc values ≤ 2, with a cumulative weight of 0.544 (Table 1). The best-supported model included four predictors: species, SD, perch category, and distance to the nearest built-up area. Distances to the nearest dirt road and asphalt road were excluded from further analyses. The final linear model (*R*^2^ = 0.605) indicated that distance to the nearest built-up area was negatively associated with FID, while SD had a positive effect (Table 2, Fig. 3). The Brown Shrike exhibited a significantly greater FID than the Red-backed Shrike (Fig. 4). Perch category did not significantly influence FID.

**Table 1:** Ranking of the best candidate models describing the influence of habitat parameters on the flight initiation distance (FID) of two shrike species.

Fixed effects	df	LL	AICc	ΔAICc	AICc wt
Intercept+Build+SD+Species+Perch	8	−77.656	172.2	0.00	0.239
Intercept+Build+SD+Species	5	−81.117	172.6	0.40	0.196
Intercept+Build+SD+Species+Perch+Asph	9	−77.324	173.8	1.57	0.109

**Notes.**

df Degrees of freedom LL model log-likelihood AICc corrected Akaike Information Criterion ΔAICc the difference between the model and the best-supported model and model weight (AICcwt) are shown

**Table 2:** Estimates of linear model coefficients for factors affecting FID. The reference levels were “Red-backed Shrike” for Species and “Fence” for Perch category.

Parameters	Estimate	*SE*	*t*-value	*P*-value
Intercept	2.590	0.093	27.854	<0.001
Build	−0.141	0.057	−2.486	0.014
SD	0.010	0.001	9.866	<0.001
Species: Brown Shrike	0.595	0.066	9.053	<0.001
Perch: Fence	0.108	0.083	1.302	0.195
Perch: Line	−0.161	0.114	−1.410	0.161
Perch: Tree	−0.073	0.072	−1.017	0.310

## Discussion

Our study demonstrates that proximity to human settlements is a key factor differentiating the behavioral responses of the two compared species. In Laos, Brown Shrike perching sites were located significantly farther from built-up areas than those of Red-backed Shrikes in Poland. Furthermore, FID differed between the species, with Red-backed Shrikes flushing at shorter distances than Brown Shrikes. This pattern supports our prediction that birds in regions with lower hunting pressure respond less intensely to human presence—remaining closer to settlements and delaying flight—compared to species in areas with higher levels of human exploitation. Unlike in many European countries, such as Poland, villagers in Laos traditionally hunt birds for subsistence (Hansel, 2004), and various passerine species are commonly found in local markets (Xayyasith, Douangboubpha & Chaiseha, 2020). Hunting can directly reduce population size and alter demographic structures through the selective removal of individuals, potentially leading to long-term demographic and evolutionary consequences (Grzegorczyk et al., 2024). Studies on game birds have shown that FID increases significantly in populations exposed to hunting, reflecting heightened antipredator responses under lethal risk (Sooth, Nielsen & Storch, 2026). Moreover, hunting-related disturbance may increase perceived predation risk, leading to earlier escape responses and shifts in habitat or perch use, which can ultimately affect foraging efficiency and fitness (Yuan et al., 2024; Beatty, Huck & Buderman, 2024). Conversely, individuals in areas with limited hunting pressure tolerate closer human approach, reflecting behavioral adjustments or selection for reduced wariness in low-risk environments (Madsen, 1998; Fox & Madsen, 1997). On the other hand, reduced FID is a common strategy in habitats with frequent but non-lethal human activity and may be linked to greater ecological plasticity and learning ability (Piratelli et al., 2015). Repeated non-lethal exposure to humans often leads to reduced FID through habituation, although such adjustments vary across populations and environmental contexts (Blumstein, 2016). Thus, shortened FIDs in human-dominated landscapes do not necessarily indicate a lack of risk, but rather a shift in perceived risk, highlighting the role of plasticity in mediating responses to anthropogenic disturbance (Møller, 2008).

Evidence of plasticity in response to actual human threats is provided by findings from a study of Brown Shrikes in Beijing, China, where the average FID outside the breeding season was several times lower than in our study (Yin et al., 2023). Because shrikes there occurred predominantly in ruderal urban and peri-urban habitats, where direct persecution (*e.g.*, shooting) is unlikely due to the constant presence of people, their short FID suggests a high tolerance for human approach under low-risk conditions. In contrast, they increase their escape distance where strong persecution occurs, as observed in Laos. This pattern reinforces the view that variation in FID is context-dependent rather than strictly species-specific, reflecting local levels of human pressure and perceived risk rather than intrinsic behavioral differences. FID is also strongly influenced by habitat structure, particularly vegetation cover and the availability of refuges, which shape risk perception and escape decisions; birds tend to initiate flight earlier when the distance to suitable refuge is greater or when structural cover is limited (Morelli et al., 2022). Simultaneously, the degree of human disturbance, expressed as the presence and movement of people, modulates antipredator responses, with repeated exposure often leading to increased tolerance and reduced FID in human-dominated environments (Samia et al., 2015; Li et al., 2025). Consequently, even low-risk, non-lethal human activity can alter habitat and microhabitat selection, as individuals adjust escape strategies based on both habitat features and the predictability of human encounters (Morelli et al., 2022; Samia et al., 2015).

Our results revealed clear patterns, despite the fact that the comparative framework may involve certain limitations. The two species were studied in different geographic regions and at different stages of their annual cycles: Brown Shrikes during the wintering period (or early spring migration) in Laos, and Red-backed Shrikes during the breeding season in Poland. Seasonal stages are known to affect behavior through differences in energy demands and territoriality (Weathers & Sullivan, 1993; Class & Moore, 2011), while geographic variation may introduce spatial heterogeneity in predator communities and human pressure (Tryjanowski & Reino, 2004). During the breeding season, antipredator behaviour is shaped by trade-offs between adult survival and current reproductive investment, and escape decisions may vary depending on the perceived risk to both adults and offspring (De Jong, Magnhagen & Thulin, 2013). Individuals engaged in reproduction may tolerate closer approach under some circumstances, whereas elevated threat levels can simultaneously promote stronger vigilance and escape responses. In contrast, during the non-breeding season, migratory birds often adjust risk-taking behaviour to changing ecological conditions and survival demands across the annual cycle (Mikula et al., 2018). Seasonal variation in behavioral plasticity and risk allocation may therefore partly contribute to the differences observed between wintering Brown Shrikes in Laos and breeding Red-backed Shrikes in Poland. Such caveats align with broader cautions against overinterpreting findings based on only two species and two study sites (Sparks & Tryjanowski, 2005). Nonetheless, the close phylogenetic affinity, similar morphology, and comparable foraging strategies of these shrikes (Zhang et al., 2007; Lefranc, 2022) support their use as a model system for exploratory comparisons. We acknowledge, however, that morphological similarity does not necessarily imply behavioral equivalence, and species-specific responses may still arise from differences in ecological context or life-history traits. Furthermore, the present study does not fully disentangle the mechanisms underlying these behavioral differences; the observed patterns are likely shaped by a combination of local human pressure, habitat structure, seasonal context, and behavioral flexibility. Thus, our results should be interpreted as evidence of context-dependent behavioral divergence rather than a direct test of a single causal mechanism.

We also detected a negative relationship between FID and distance to the nearest built-up area. Although in many other bird species FID is shorter in urbanized and disturbed habitats (Møller, 2008; McGiffin et al., 2013; Samia et al., 2015; Samia et al., 2017; Cieśluk & Kasprzykowski, 2024), our findings are consistent with our expectations for this specific context. In Laos, areas closer to villages are visited more frequently by hunters (Xayyasith, Douangboubpha & Chaiseha, 2020), increasing the likelihood of birds being trapped or hunted for food and trade. This likely explains why Brown Shrikes flushed at greater distances near settlements. Frequent encounters with humans who pose a direct threat could further reinforce such avoidance behavior. However, stronger escape responses carry ecological costs: increased FID and frequent disturbances may reduce foraging efficiency, lower survival, and decrease reproductive success, potentially contributing to population declines (Fernández-Juricic et al., 2006; Møller, 2008; Piratelli et al., 2015). In some species, such as urban Hooded Crows (*Corvus cornix*), FID increases during the breeding season, reflecting heightened risk aversion when caring for offspring (Novčić & Parača, 2022). Shrikes are well known for their aggressive nest defense, but in the Red-backed Shrike, this behavior intensifies significantly only when large chicks or fledglings are present (Tryjanowski & Goławski, 2004). Our observations were conducted at the very beginning of the breeding season, when territorial aggression and nest-defense behavior remain minimal. Thus, the timing of fieldwork likely reduced any potential bias related to breeding-stage effects. Consequently, the longer FID recorded during wintering in Laos relative to breeding in Poland suggests that differences in human disturbance significantly influence shrike reactions to human approach.

The effect of proximity to roads on the alertness of shrikes was not significant. For both species, the distances of perching sites from the nearest dirt and asphalt roads were similar. Roads, particularly local and unpaved ones, may function as open feeding grounds, providing the visibility required for efficient prey detection (Morelli, 2013; Morelli et al., 2015; Hamza & Hanane, 2021). This aligns with the shrike’s ecology as a “sit-and-wait” predator (Severinghaus & Liang, 1995; Yosef, 2004). Suitable foraging habitats near roads appear to be utilized in a similar manner by both species (Golawski & Meissner, 2008).

Finally, we found a positive relationship between SD and FID, a pattern documented in various other bird species (Blumstein, 2003; Weston et al., 2012; Mikula et al., 2022; Kasprzykowski & Golawski, 2025). This correlation is expected, as distance and time are closely linked when a human approaches at a constant speed (Tryjanowski et al., 2020; Golawski, Charalambidou & Golawska, 2023). However, these results should be interpreted as patterns of perch-site use rather than strict selection, as the availability of alternative perches was not quantified. Therefore, observed differences may reflect not only responses to human infrastructure but also variation in prey abundance, vegetation structure, visibility, and predator pressure (Yosef, 2004; Morelli et al., 2015; Golawski, Kasprzykowski & Al Sariri, 2020).

## Conclusions

This study suggests that proximity to human settlements is a major factor shaping habitat use and antipredator behavior in shrikes exposed to different levels of anthropogenic pressure. Differences in perching-site choice and FID between Red-backed Shrikes in Poland and Brown Shrikes in Laos indicate that behavioral responses to humans are primarily context-dependent, reflecting local hunting pressure rather than intrinsic species-specific traits. Birds in areas with lower exploitation tolerated closer human presence and delayed escape, whereas those exposed to higher persecution exhibited stronger avoidance and greater vigilance. The negative relationship between FID and distance to built-up areas suggests that perceived human risk plays a key role in shaping escape responses, particularly where settlements are associated with hunting activity. Although the comparison involved different geographic regions and seasonal stages, the consistency of the observed patterns implies that variation in human pressure may outweigh potential phenological effects. Overall, our results highlight the value of behavioral indicators such as FID for understanding avian responses to human disturbance and for informing conservation strategies in human-modified landscapes.

##  Supplemental Information

10.7717/peerj.21506/supp-1 Supplemental Information 1 Raw data

10.7717/peerj.21506/supp-2 Supplemental Information 2 Raw data codebook

10.7717/peerj.21506/supp-3 Supplemental Information 3 Appendix and Supplemental Figures

## Additional Information and Declarations

### Data Availability

The following information was supplied regarding data availability: The data is available in the Supplemental Files. The data is available at Mendeley: Kasprzykowski, Zbigniew (2026), “perch-site selection in shrikes: effects of human disturbance in Poland and Loas”, Mendeley Data, V1, doi: 10.17632/f8gm2zgk4m.1.
