# Supplementary material for: Escape response and perch-site choice in shrikes: effects of human disturbance in Europe and Southeast Asia
Source: PeerJ. 2026 Jun 30;14:e21506. doi: 10.7717/peerj.21506 (PMC13330747; doi:10.7717/peerj.21506)
Supplement: Supplemental Information 2 [file peerj-14-21506-s002.docx]

Raw data legend
id - identification number

spec – species: col (Red-backed Shrike *Lanius collurio*) and cri (Brown Shrike *Lanius cristatus*)

sd - starting distance

high- perch height

perch - perch category: fence, bush, tree, and power line (line).

fid - flight initiation distance

road - distances (km) from the perching site to the nearest dirt road

asph - distances (km) from the perching site to the nearest asphalt road

build - distances (km) from the perching site to the nearest built-up area
