# Supplementary material for: Escape response and perch-site choice in shrikes: effects of human disturbance in Europe and Southeast Asia
Source: PeerJ. 2026 Jun 30;14:e21506. doi: 10.7717/peerj.21506 (PMC13330747; doi:10.7717/peerj.21506)

Appendix 1

Table S1. Mean and SE of the variables used to analyse differences in habitat choice by the Red-backed Shrike and the Brown Shrike in relation to roads and human settlements.

| Variables | Red-backed Shrike (N=102) | Brown Shrike (N=69) | Total (N=171) |
| --- | --- | --- | --- |
| Road (km) | 0.11±0.01 | 0.08±0.01 | 0.09±0.01 |
| Asph (km) | 0.55±0.06 | 0.63±0.07 | 0.58±0.04 |
| Build (km) | 0.75±0.05 | 1.07±0.07 | 0.88±0.04 |
| SD (m) | 66.17±28.79 | 83.49±31.31 | 73.16±2.37 |
| FID (m) | 26.35±1.35 | 53.73±24.43 | 37.40±1.76 |

Figure S1. A sample map showing the distances from the perching site to the nearest dirt road (yellow), the nearest asphalt road (red), and the nearest built-up area (blue) in two study sites. Background imagery from Google Maps. Map data © Google, Airbus, 2025.  
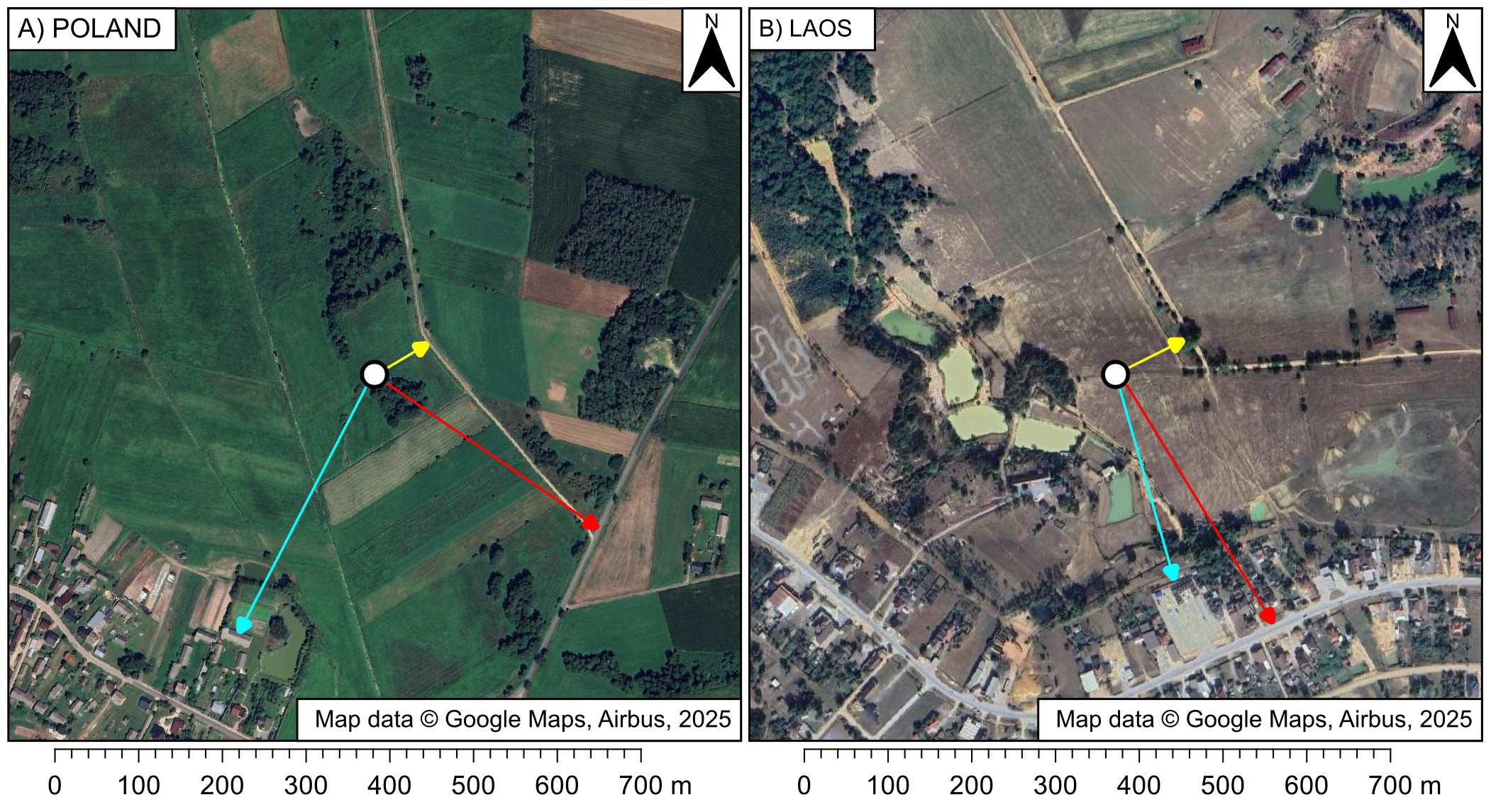

Supplement: Supplemental Information 3 [file peerj-14-21506-s003.docx]
